# Breast Radiotherapy (RT) Using Tangential Fields (TgF): A Prospective Evaluation of the Dose Distribution in the Sentinel Lymph Node (SLN) Area as Determined Intraoperatively by Clip Placement

**DOI:** 10.1245/s10434-014-3966-1

**Published:** 2014-08-06

**Authors:** Yazid Belkacemi, Veronique Bigorie, Qiong PAN, Ryan Bouaita, Frederic Pigneur, Emmanuel Itti, Hakima Badaoui, Elias Assaf, Philippe Caillet, Elie Calitchi, Romain Bosc

**Affiliations:** 1 AP-HP, GH Henri Mondor. Service d’Oncologie-Radiothérapie et Centre Sein Henri Mondor. Université Paris-Est Créteil (UPEC), Créteil, France; 2 APHP, GH Henri Mondor. Service de Chirurgie Plastique et Centre Sein Henri Mondor. Université Paris-Est Créteil (UPEC), Créteil, France; 3 APHP, GH Henri Mondor. Service d’Imagerie Médicale et Centre Sein Henri Mondor, Créteil, France; 4 APHP, GH Henri Mondor. Service de Médecine Nucléaire et Centre Sein Henri Mondor. Université Paris-Est Créteil (UPEC), Créteil, France; 5 APHP, GH Henri Mondor. Service d’Anatomo-Pathologie et Centre Sein Henri Mondor, Créteil, France; 6 APHP, GH Henri Mondor. Service d’Oncologie Médicale et Centre Sein Henri Mondor, Créteil, France; 7 APHP, GH Henri Mondor. Unité d’Oncogériatrie, Créteil, France

## Abstract

**Background:**

Randomized trials have established that patients with limited involvement of sentinel lymph node (SLN) do not require axillary lymph node dissection (ALND). The similar outcome in patients with ≤2 positive SLN with or without additional ALND is attributed, in part, to tangential fields (TgF) RT. We evaluated the dose distribution in the SLN biopsy area (SLNBa) as determined intraoperatively by clips placement for radiotherapy (RT) optimization.

**Methods:**

This prospective study included 25 patients who had breast conservation. Titanium clips were used intraoperatively to mark the SLNBa. All patients had 3D-conformal RT using standard (STgF) or high tangential fields (HTgF). Axillary levels, SLNBa, and organs at risk were contoured on a CT scan. Dose distribution and overlap between TgF and target volumes were analyzed.

**Results:**

The average doses delivered to axilla levels I-III and SLNBa were 25, 5, 2, and 33 Gy, respectively. The average dose delivered to SLNBa was higher using HTgF with better coverage of the axilla. Only 12 of 25 patients (48 %) had their SLNBa completely covered by the TgF. There was no impact of TgF size on ipsilateral lung dose. The mean heart dose delivered using STgF was lower than HTgF.

**Conclusions:**

In the era of SLNB, axilla and SNLBa RT technique has to be standardized to deliver adequate dose. We recommend the use of HTgF or direct axillary RT techniques (such as in AMAROS trial) in patients with metastases in SLN without ALND completion, when only TgF are expected to cure potential residual disease in the axilla.

An extensive literature, including seven randomized trials and B32 trial update at 10 years, has established that axillary lymph node dissection (ALND) is not required in patients with negative sentinel lymph node biopsy (SLNB) in which axillary recurrence is rare.^1^,^2^ SNLB represents the standard procedure for patients with early breast cancer (BC) and clinically node-negative (cN0). Thus, Saint-Gallen guidelines state that ALND should not be completed in cN0 patients with one to two macrometastatic (MAC) in the SLNs after breast-conserving surgery (BCS) and tangential field (TgF) radiotherapy (RT).^3^ The American Society of Clinical Oncology (ASCO) updated guidelines concluded recently that women with one to two metastatic SLNs planning to undergo BCS with whole breast radiation therapy (WBRT) should not undergo ALND.^4^

Moreover, in the ACOSOG Z0011 trial, 6-year outcome after BCS plus WBRT was equivalent in SLNB and ALND patients with ≤2 positive SLNs. This equivalence was attributed to the potential cure of axillary residual disease with systemic therapy and TgF RT.^5^ While radiation parameters and dose distribution in the axilla were not reported in the initial publication, Jagsi et al. tried recently to detail radiation treatments from the 605 available RT report forms.^5^ No clear conclusions could be drawn from the analyses on whether additional regional nodal RT was necessary or beneficial for these patients.^6^ The utility of TgF RT has been established as the standard of care. The issue that remains outstanding relates to the benefit to include the lymphatics.

Our study was undertaken to determine the dose distribution in the sentinel lymph node biopsy area (SLNBa) marked intraoperatively by clips. This could be helpful for RT optimization when only TgF are used for WBRT in patients with SLN involvement without ALND completion.

## Methods

This prospective study included 25 patients who have undergone BCS in a single institution between April 2012 and March 2013. The Henri Mondor Breast Center Multidisciplinary Committee has approved the protocol. Patients’ characteristics are presented in Table 1.

**Table 1:** Patients’ characteristics

Median age (range) (year)	61 (43–84)
Clinical tumor classification	
Tis	1
T1a	4
T1b	8
T1c	12
Pathologic nodal status	
pN0	23
pN1 (mi)	1
pN1a	1
Hormone receptor and Her 2 status	
Hormone receptors +	23
Hormone receptors −	2
HER2 positive	2
Tumor differentiation	
Grade I	9
Grade II	15
Pathology	
Ductal invasive carcinoma	22
Lobular invasive carcinoma	2
Carcinoma in situ	1
RT parameter	
Total dose (Gy)	
With boost (60–66 Gy)	21
Without boost (40–50 Gy)	4
Median delay from BCS–RT (range) (day)	43 (13–50)
Patient morphology	
Mean weight (range) (kg)	67 (47–102)
Mean size (cm)	164
BSA (m^2^)	1.74 (1.46–2.17)
Tangential fields thickness (cm)	13.6 (9–18)

*BCS* breast conserving surgery; *RT* radiotherapy; *BSA* body surface area

### Surgery Procedure

All patients underwent BCS and SLNB procedure for invasive BC (tumor size <3 cm, cN0). SLNB mapping was performed using technetium-99 m-labelled human albumin colloid particles. Blue dye was not systematically administered. SLNs were identified with a gamma detecting probe and/or blue dyed. After SLNs removal two titanium clips were placed to mark the location. No ALND was performed in this study.

### Radiation Therapy Technique

All patients had 3D-conformal RT. Two radiation oncologists contoured axilla nodal volumes, SLNBa, and organs at risk using the RTOG contouring atlas.^7^ The WBRT technique and indications followed the French guidelines described elsewhere.^8^ Height of TgF was defined individually to target the breast volume. This study evaluated SLNBa coverage by TgF as determined intraoperatively by clips placement. For height TgF analyses, STgF was defined with the superior border set at 2 cm below the humeral head, whereas HTgF consisted of a superior border placed at the inferior edge of the humeral head.

**Fig. 1:**
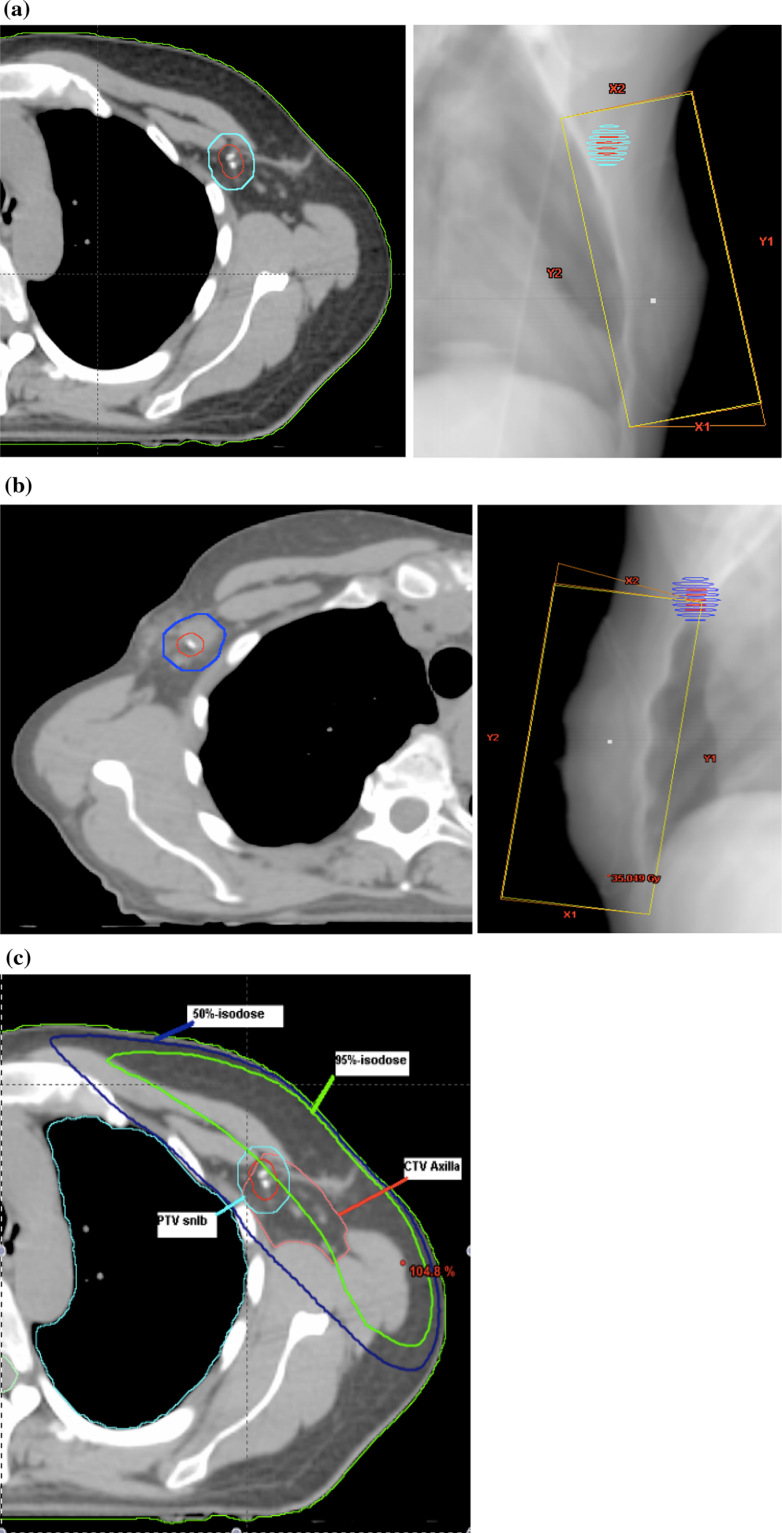
Topographic distribution of the clip locations, volumes and isodoses (95 and 50 %). **a** Example of clips topography in the sentinel lymph node biopsy area without associated seroma and tangential fields including totally the PTV_SLNB_. *CTV*_*SLNB*_ clinical target volume of the sentinel lymph node biopsy area (in *red*); *PTV*_*SLNB*_ planning target volume of the sentinel lymph node area (in *light blue*). **b** Example of clips topography in the sentinel lymph node biopsy area with associated seroma and tangential fields including partially the PTV_SLNB_. TgF were not adjusted to include totally the SLNBa in patients with negative SLN status. *CTV*_*SLNB*_ clinical target volume of the sentinel lymph node biopsy area (in *red*); *PTV*_*SLNB*_ planning target volume of the sentinel lymph node area (in *blue*). **c** Example of PTV_SLNB_ and axilla levels CTV coverage by 95 and 50 % isodoses using standard tangential fields

The SLNBa was defined as a clinical target volume (CTV_SLNB_) with 5 mm in diameter surrounding the clips. To account for position uncertainties, we defined SLNBa planning target volume (PTV_SLNB_) with a 10 mm extension around the CTV_SLNB_ (Fig. 1). Only two patients had seroma in SLNBa with a maximum diameter of 24 and 10 mm. In both cases, clips were not displaced by the seroma cavity. The latter was included in the PTV_SLNB_ (Fig. 1).

**Fig. 2:**
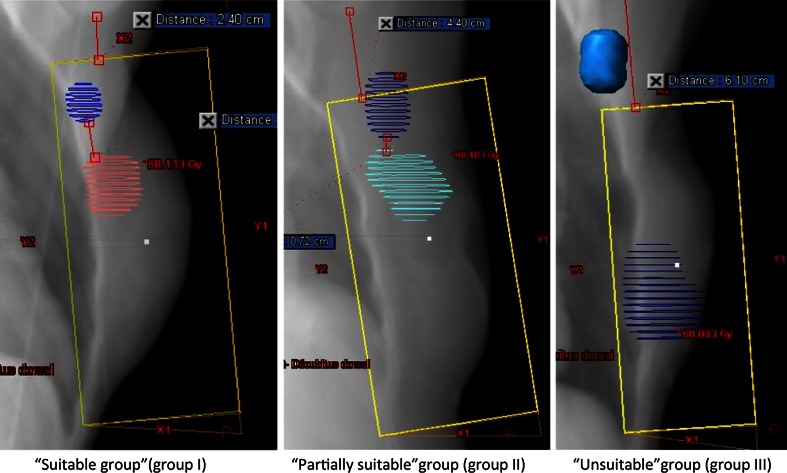
Coverage of the sentinel lymph node biopsy area by tangential fields. Three groups of TgF-PTV_SLNB_ overlap were defined: 100 % overlap (“suitable group” with PTV_SLNB_ completely included in the TgF), ≥50 % overlap (“partially suitable group” with PTV_SLNB_ partially included in the TgF), and 0–49 % overlap (“unsuitable group” with 50 % of the PTV_SLNB_ or completely outside the TgF). Average dose was 46, 34, and 8 Gy, respectively in the three groups

Dose-volume-histograms were analyzed according to axilla volumes receiving 95 % (V95) or 50 % (V50) of the prescribed dose. All values were compared according to the use of STgF (*n* = 20) or HTgF (*n* = 5). Overlaps between the TgF and the PTV_SLNB_ were analyzed in three groups of TgF-PTV_SLNB_ overlap percentages: 100 % overlap (“suitable group”; PTV_SLNB_ completely in the TgF), ≥50 % overlap (“partially suitable group”; PTV_SLNB_ partially in the TgF), and 0–49 % overlap (“unsuitable group”; ≤49 % of the PTV_SLNB_ or completely outside the TgF; Fig. 2). Dose distribution was calculated in each of the three groups.

### Statistical Analyses

All comparisons and correlations were performed using *t* tests using SPSS software. Multiples comparisons were analyzed using an ANOVA post hoc Bonferroni. The level of significance was stated at *p* < 0.05.

## Results

The median number of harvested SLNs was 1 (average 1.6; range 1–6). One patient had micrometastasis (MIC; >0.2–2 mm) and one had 1 MAC out of 4 SLNs, respectively. Adjuvant systemic therapy and WBRT parameters are presented in Table 1.

### Dose and Volume Coverage

The average doses delivered to axillary levels I, II, III, and SLNBa were 25, 5, 2, and 33 Gy, respectively. Whereas coverage of these four volumes by the 95 % isodose was limited (0–4 %), the 50 % isodose covered 47, 4, 1, and 65 %, respectively (Table 2). The average doses delivered to these four volumes were higher using HTgF than STgF (38 vs. 22 Gy, *p* = 0.004; 11 vs. 3 Gy, *p* = 0.019; 5 vs. 2 Gy, *p* = 0.003; 45 vs. 30 Gy, *p* = 0.02), respectively (Table 3). While average D50 were higher in HTgF versus STgF patients, no difference was observed for D95. The results are presented in Table 3.

**Table 2:** Dose distribution in axilla levels I to III and the sentinel lymph node biopsy area

RT parameters	Targets	Average (range)		
Dose (Gy)	Level I	25 (0–44)		
	Level II	5 (0–31)		
	Level III	2 (0–16)		
	PTV_SLNB_	33 (1–60)		
D95 (Gy)	Level I	5 (0–36)	V95 (%)	2 (0–23)
	Level II	1 (0–3)		0
	Level III	1 (0–2)		0
	PTV_SLNB_	25 (0–59)		4 (0–99)
D50 (Gy)	Level I	30 (1–49)	V50 (%)	47 (0–96)
	Level II	4 (0–48)		4 (0–59)
	Level III	2 (0–7)		1 (0–21)
	PTV_SLNB_	33 (1–60)		65 (0–100)

*RT* radiotherapy; *D95* dose delivered to 95 % of the target; *V95* volume of the target receiving 95 % of the prescribed dose; *D50* dose delivered to 50 % of the target; *V50* volume of the target receiving 50 % of the prescribed dose; *PTV* planning target volume; *PTV*_*SLNB*_ PTV of the sentinel lymph node biopsy area

**Table 3:** Dose distribution comparison in levels I to III and the sentinel lymph node biopsy area according to tangential fields height

RT parameters	Mean values			*p* value
	Axilla contents	STgF	HTgF	
Average dose (Gy)	Level I	22	38	0.004
	Level II	3	11	0.019
	Level III	2	5	–
	PTV_SLNB_	30	45	0.02
D95	Level I	5	6	
	Level II	1	2	NS
	Level III	1	1	
	PTV_SLNB_	22	33	
D50	Level I	26	45	<0.001
	Level II	2	12	<0.001
	Level III	1	3	–
	PTV_SLNB_	54	65	0.001

*D95* dose delivered to 95 % of the target; *D50* dose delivered to 50 % of the target; *PTV* planning target volume; *PTV*_*SLNB*_ PTV sentinel lymph node biopsy area; *STgF* standard tangential fields; *HTgF* high tangential fields; *NS* not significant

### SLNB Area Coverage

In the STgF group (*n* = 20), the coverage of SLNBa by the TgF was “suitable” in eight cases (40 %), “partially suitable” in six cases (30 %), and “unsuitable” in six cases (30 %). In the HTgF group, four and one patients were considered “suitable” or “partially suitable,” respectively. Finally, the SLNBa was completely covered by the TgF in 12 of 25 patients (48 %), independent of the TgF size. In the two patients with involved SLNs, STgF were modified as HTgF to include totally the SLNBa.

The average dose delivered to the PTV_SLNB_ was lower in the “unsuitable” (8 Gy) versus “partially suitable” (34 Gy) versus the “suitable” group (46 Gy; *p* = 0.01). The difference also was significant in terms of the average D95 (*p* = 0.017) and D50 (*p* = 0.028) delivered to the PTV_SLNB_ (Table 4).

**Table 4:** Dose distribution comparison in axilla contents of patients groups determined regarding the overlap between the sentinel lymph node biopsy area and tangential fields

RT parameters	Axilla contents	“Suitable” group (G I)	“Partially suitable” group (G II)	“Unsuitable” group (G III)	*p* value
	n	*12*	*7*	*6*	
Average dose (Gy)	Level I	31	29	8	NS
	Level II	8	2	1	0.027
	Level III	4	1	1	NS
	PTV_SLNB_	46	34	8	0.01
D95	Level I	8	3	0	0.03
	Level II	1	1	0	NS
	Level III	1	1	0	–
	PTV_SLNB_	42	14	1	0.017
D50	Level I	42	32	3	0.045
	Level II	7	2	1	–
	Level III	2	2	1	–
	PTV_SLNB_	47	31	7	0.028

*D95* dose delivered to 95 % of the target; *D50* dose delivered to 50 % of the target; *PTV* planning target volume; *PTV*_*SLNB*_ PTV sentinel lymph node biopsy area; *NS* not significant

### Organs at Risk Analyses: Ipsilateral Lung and Heart

The percentage of ipsilateral lung volumes receiving 5 Gy (V5), 10 Gy (V10), 20 Gy (V20), and the average dose were calculated. There was no statistical difference between HTgF versus STgF patients for: V20 (7 vs. 6 %; *p* = 0.33), V10 (13 vs. 10 %; *p* = 0.33), and V5 (26 vs. 20 %; *p* = 0.33). The mean ipsilateral lung dose using HTgF was not significantly greater than STgF (6 vs. 5 Gy; *p* = 0.2). In the left BC patients, the mean heart dose was higher with HTgF versus STgF (2.6 vs. 1.4 Gy; *p* = 0.02).

## Discussion

Our group showed recently that STgF planed for breast RT does not allow adequate coverage of the axilla.^9^ These findings are important to consider in the context of the international guidelines on ALND avoidance when systemic therapy and RT are expected to cure potential residual disease in the axilla.^1^–^5^ However, in the past decade, several studies have shown that STgF fails to adequately treat levels I–II.^1^,^9^–^15^ In this context, two major points on optimal regional RT technique to cover the axilla correctly should be considered. First, recent data from randomized trials and a meta-analysis have shown that nodal RT increases overall survival and particularly distant metastases free-survival.^16^–^18^ Second, RT to the axilla has been shown as safe and equivalent to ALND in patients with MIC in SLNB.^19^ These results highlight the importance of redefining adjuvant nodal RT in the SLNB era.

There is a direct relationship between prognosis and the number of involved LNs. In patients with positive SLN, the percentage of the SLN occupied by tumors and the number of SLNs removed are independently predictive of non-SLN involvement. In addition, the non-SLN involvement negatively influences survival.^20^ Reed et al. reported that none of 13 patients with ITCs who underwent an ALND had additional positive nodes compared with 27 % of patients with MIC. At 5 years, distant recurrence rates in SLN-negative, isolated tumor cells, MIC, and MAC groups were 6, 8, 14, and 21 %, respectively. The presence of MIC in the SLN was associated with a significantly shorter disease-free interval than was SLN negativity (*p* < 0.02).^21^

The RT objectives in case of axilla residual disease are to reduce the risk of locoregional recurrence and to prevent distant metastases from axillary sanctuary as hypothesized by Hellman: “RT is stopping metastases at their source.” 22 These two objectives are highly linked together and to the axilla contents dose. Francissen et al. reviewed 16 studies describing patients with MAC disease in the SLN without ALND completion.^23^ After 43 months of follow-up, they observed only 24 axillary recurrences out of 3,268 (0.7 %) among whom three received RT.^23^ The axillary recurrence rate is even lower in the database study by Yi et al. with only 0.1 % among the 1, 473 patients with MAC in the SLN.^24^ Other smaller cohorts studies showed rates between 0 and 7.1 %.^1^ However, there is a lack of RT technique data to conclude on the relationship between axilla underdosage and local recurrence.^2^,^5^ In our study, STgF coverage of SNLBa was complete (“suitable” group) in only eight cases (40 %), whereas HTgF covered the SLNBa in four of five (80 %) patients. Indeed, the significant variations of the anatomical location of the SLNs do not allow coverage of the SLNBa with STgF, which does not include the LNs at highest risk of containing tumor. Therefore, some authors suggested removing the superior-posterior corner multileaf collimators of the TgF to cover axilla levels.^13^,^25^,^26^ In our study, STgF have been modified as HTgF to include the whole SLNBa in both patients with metastases in the SLN.

Tumoricidal radiation dose also should be questioned. The evaluation of the delivered doses to axilla contents in the Z0011 study remains uncertain.^5^ The recent report from Jagsi et al. based on a centralized review of 228 (out of 605) patients provided only partial results on RT technique. Among the 185 (out of 228) patients with TgF-only treatment there was sufficient data to evaluate TgF height in 142 (76.8 %) patients. Because RT parameters and nodal volumes details were lacking, they could not evaluate the dose distribution in the axilla. However, they showed that direct nodal irradiation technique was mainly used in case of multiple nodal involvements for better coverage of the axilla compared with the HTgF technique.^6^

Several reports have highlighted that axillary nodal coverage depends on the upper TgF border. Studies using STgF showed that only approximately 50 % of level I and 20–30 % of level II nodes might receive 95 % of the prescribed dose.^10^,^26^–^28^ However, several of these early studies used conventional simulation with surgical clips as anatomic landmarks to evaluate the dose distribution. For example, Reed et al. showed that STgF fail to treat the axillary level I–II anatomic volume adequately, with approximately 50 % receiving a therapeutic dose. They concluded that surgical clips from ALND grossly underestimate the level I–II axilla nodal volume and should not be relied on for therapy planning.^29^

The use of HTgF in patients with MIC in the SLN is an important issue to consider when no ALND is indicated. In the Z0011 trial, HTgF were used in 50 and 52.6 % of patients randomized to the ALND and SLNB arms, respectively. Of note, only 43 (19 %) patients received direct regional RT using ≥3 fields. In this small group receiving a third field, there was a trend suggesting that treatment with posterior axillary boost field was more common in patients who had SLNB alone (12/21 vs. 6/22; *p* = 0.0066) and those receiving nodal RT had greater number of LNs involved (*p* < 0.001).^6^ Axilla coverage may be paramount to locoregional control and to decreasing the risk of metastatic dissemination from residual uncontrolled disease in the axilla. This is particularly important to consider regarding the recent data on overall and metastatic free-survival benefits from nodal RT in large clinical trials.^16^–^18^

Our study was undertaken to address the question of dose distribution and SLNBa coverage according to TgF size. We showed that the SLNBa was completely covered by the TgF independently from its size in only 48 % of the patients. The average dose in the SLNBa was 33 Gy. However, there was significantly higher delivered dose using HTgF. The average dose is considered as nontumoricidal in at least the partially suitable (34 Gy) and unsuitable (8 Gy) groups (Table 4). To overcome the underdosage of axilla, some authors have suggested tailoring TgF to targets. Kiel et al. recommended that the cranial field edge should be 1.2 cm below the humeral head and that 2.5 cm of the lung be included in the breast TgF to adequately cover the axilla.^27^ Schlembach et al. demonstrated that the LNs at greatest risk are 2 cm below the humeral head in 95 % of cases.^26^ Reznik et al. reported an increase of 20–30 % of the average dose delivered to axilla levels when using HTgF.^10^ Considering the same borders, these values were lower in our earlier study in which STgF was used in the majority of patients.^9^

Many authors have attempted to define the anatomical borders by surgically marked axilla volumes and evaluated dose distribution at each level. Krasin et al. showed that only 1 of 25 patients received 50 Gy in the Level I of the axilla, and no patient had an adequate coverage of the Level II–III.^11^ Reed et al. showed a significant volume difference between the anatomical and the surgically marked axillary volumes in 18 of 50 patients undergoing ALND with more adequate coverage of the axilla in the latter.^29^ Another way to define marked axilla is to use a sentinel clip at the caudal border in the anatomically defined axilla. Using this procedure, Orecchia et al. showed that only 1 of 15 patients received 40 Gy in the axilla in a context of significant volume reduction.^15^

In a context of the ALND avoidance in selected patients, the debate on the axilla underdosage by TgF has to be addressed as the risk of non-SLNs involvement may depend on the anatomic location of SLN and its degree of involvement.^30^ The latter as determined by conventional histology has been described as a predictive factor for additional axillary involvement.^21^ Thus, as studies that have been undertaken to quantify intraoperatively the total tumor load in the positive SLNs showed that it is possible to predict additional non-SLN metastasis in the axilla with a high specificity, the authors suggested that this could be used to guide decisions for ALND completion.^30^ From the radiation oncology view, for an adequate coverage of the axilla, the use of direct fields could be considered rather than TgF in the patients with SLN involvement without further ALND.^19^

## Conclusions

In patients undergoing BCS followed by WBRT, STgF provide a limited coverage of the axilla contents and deliver a nontumoricidal dose to potential axillary residual disease. The RT technique to deliver adequate dose to axilla and SLNBa has to be standardized with the use of HTgF or direct fields as described in AMAROS trial.^19^ This is true, insofar as we have: only limited follow-up in the SLNB trials without RT technique details, no clear tumoricidal dose level for residual disease, and uncertainties on the disease in the remained axilla non-SLN.
